# Vaccination-driven evolution of infectious bronchitis virus in Korea: implication for the control of other coronavirus infections

**DOI:** 10.3389/fvets.2026.1859772

**Published:** 2026-06-23

**Authors:** Seung-Ji Kim, Kang-Seuk Choi

**Affiliations:** 1 Laboratory of Avian Diseases, College of Veterinary Medicine, Seoul National University, Seoul, Republic of Korea; 2 Research Institute for Veterinary Science, College of Veterinary Medicine, Seoul National University, Seoul, Republic of Korea; 3 BK21 FOUR Program For Future Veterinary Medicine Leading Education and Research Center, College of Veterinary Medicine, Seoul National University, Seoul, Republic of Korea

**Keywords:** genetic recombination, immune selection pressure, infectious bronchitis virus, poultry production systems, vaccination, viral evolution

## Abstract

Infectious bronchitis virus (IBV) remains a major pathogen in global poultry production due to its extensive genetic diversity and rapid evolutionary capacity. Antigenic diversification driven by mutation, homologous recombination, and sustained immune selection pressure complicates long-term control and reduces vaccine effectiveness. Increasing evidence suggests that IBV evolution is largely driven by the selective expansion of pre-existing variants under changing ecological and immunological conditions, rather than by the *de novo* emergence of novel lineages. South Korea provides a valuable model for understanding these processes within intensive poultry systems characterized by high host density, extensive farm connectivity, widespread vaccination, and long-term molecular surveillance. Since its first isolation in 1986, IBV in Korea has undergone repeated cycles of lineage emergence, diversification, and replacement, culminating in the predominance of nephropathogenic GI-19 viruses, including KM91 and QX-like variants. Persistent co-circulation of multiple lineages, together with frequent recombination and regional viral introduction, has shaped a highly dynamic viral population. Within this environment, vaccination plays a central yet paradoxical role. While essential for disease control, widespread vaccination imposes continuous immune selection pressure and may contribute to co-circulation of vaccine-derived and field strains, facilitating recombination and the emergence of immune escape variants. These processes drive lineage turnover and antigenic mismatch between circulating viruses and vaccine strains, forming a vaccination-driven evolutionary cycle. This review integrates global IBV diversity with insights from the Korean system to propose an evolution-centered framework in which viral genetic plasticity, host population dynamics, and vaccination practices interact. These findings have important implications for optimizing vaccination strategies and provide broader insights into the evolutionary dynamics of rapidly evolving RNA viruses under sustained immune pressure.

## 1 Introduction

Infectious bronchitis virus (IBV) is a highly contagious avian coronavirus responsible for infectious bronchitis (IB) in chickens and remains one of the most significant viral pathogens affecting the global poultry industry (1, 2). Infection can lead to respiratory disease, nephritis, reduced growth performance, decreased egg production, and poor egg quality, resulting in considerable economic losses in both broiler and layer production systems (3, 4).

IBV is an enveloped virus belonging to the genus *Gammacoronavirus* within the family *Coronaviridae* and the order *Nidovirales* (1, 5). The virion is roughly spherical, with a diameter of approximately 125 nm, and is characterized by prominent spike projections on its surface that give coronaviruses their characteristic crown-like appearance (6, 7). The viral genome consists of a positive-sense single-stranded RNA of approximately 27–30 kb, one of the largest genomes among RNA viruses (7). The genome follows the canonical coronavirus organization, with the replicase genes (ORF1a/1b) spanning approximately two-thirds of the genome and encoding polyproteins that are cleaved into 15 non-structural proteins (NSPs) by two virally encoded proteases (8). The remaining one-third of the genome contains genes encoding the structural proteins and several accessory proteins. The four major structural proteins of IBV include the spike (S) glycoprotein, membrane (M) protein, envelope (E) protein, and nucleocapsid (N) protein (1, 8, 9). Among these, the S glycoprotein plays a particularly important role in viral entry and host immune recognition (10, 11). The S protein is cleaved into two subunits, S1 and S2, with the S1 subunit containing receptor-binding domains and major antigenic determinants responsible for serotype specificity and antigenic variation (10, 12, 13).

Based on the complete S1 gene sequence, IBV strains have been classified into multiple genotypes and lineages worldwide (14–16). Several lineages—including GI-1 (Massachusetts), GI-13 (793B), GI-19 (QX) and GI-23 have achieved global distribution and have been responsible for numerous IB outbreaks (15, 17–21). However, the epidemiology of IBV differs substantially among regions due to variations in poultry production systems, vaccination practices, and ecological conditions (5, 18, 22). Consequently, understanding regional IBV evolution is essential for interpreting global epidemiological patterns and improving national disease control strategies.

A number of review articles have previously summarized the molecular biology, global diversity, and vaccination strategies of IBV (1, 3, 5, 15, 18, 21, 22). However, relatively few studies have examined the long-term evolutionary dynamics of IBV within a defined national poultry production system (23–25). Such longitudinal perspectives are particularly valuable because IBV populations evolve through complex interactions among viral mutation, recombination, host immunity and production ecology as well as national-level disease control like vaccination and surveillance systems (5, 18, 22). Understanding how these factors interact over time within intensive poultry production environments can provide important insights into the mechanisms driving viral diversification and genotype turnover.

South Korea provides a particularly informative setting for examining the evolutionary dynamics within modern poultry production systems. Despite its relatively small geographic area, the Korean poultry industry is characterized by high farm density, relatively large flock sizes, and vertically integrated production structures in which hatcheries, contract farms, feed supply systems, and processing facilities are tightly connected (26–29). Such production networks create ecological conditions that allow respiratory viruses to circulate efficiently within and among livestock populations (30, 31). At the same time, IB has long been recognized as a major health concern in the Korean poultry industry, leading to the widespread and sustained implementation of vaccination programs across different production sectors (32–34). In addition to these production characteristics, South Korea has maintained continuous molecular monitoring of IBV circulating in commercial poultry farms for several decades (32, 34–44). The availability of long-term epidemiological data and virus sequence information provides a valuable opportunity to observe how IBV populations change over time within a relatively well-defined production ecosystem. Furthermore, the Korean poultry industry is closely connected to regional breeding stock supply chains and poultry production networks in East Asia, which may facilitate the regional movement of IBV strains and contribute to the genetic diversity observed within domestic poultry populations (33, 43, 45, 46).

Collectively, these characteristics make the Korean poultry industry an informative natural setting for examining how viral mutation, recombination, host immunity, and production ecology interact to shape the evolutionary trajectories of IBV populations. Insights derived from such long-term observations can therefore contribute not only to understanding the epidemiology of IBV within Korea but also to interpreting broader patterns of IBV evolution and control in modern poultry production systems worldwide. In this review, we summarize current knowledge on the global genetic diversity of IBV and provide a comprehensive overview of the molecular epidemiology and evolutionary dynamics of IBV in South Korea. Particular emphasis is placed on genotype turnover, recombination patterns, pathogenic diversity, and vaccination strategies associated with nephropathogenic GI-19 IBV strains. By integrating long-term epidemiological observations with recent molecular studies, this review aims to highlight how the Korean experience can provide broader insights into the mechanisms driving IBV evolution and inform future strategies for global IB control.

## 2 Replication strategy of IBV and its implications for viral evolution

**Figure 1:**
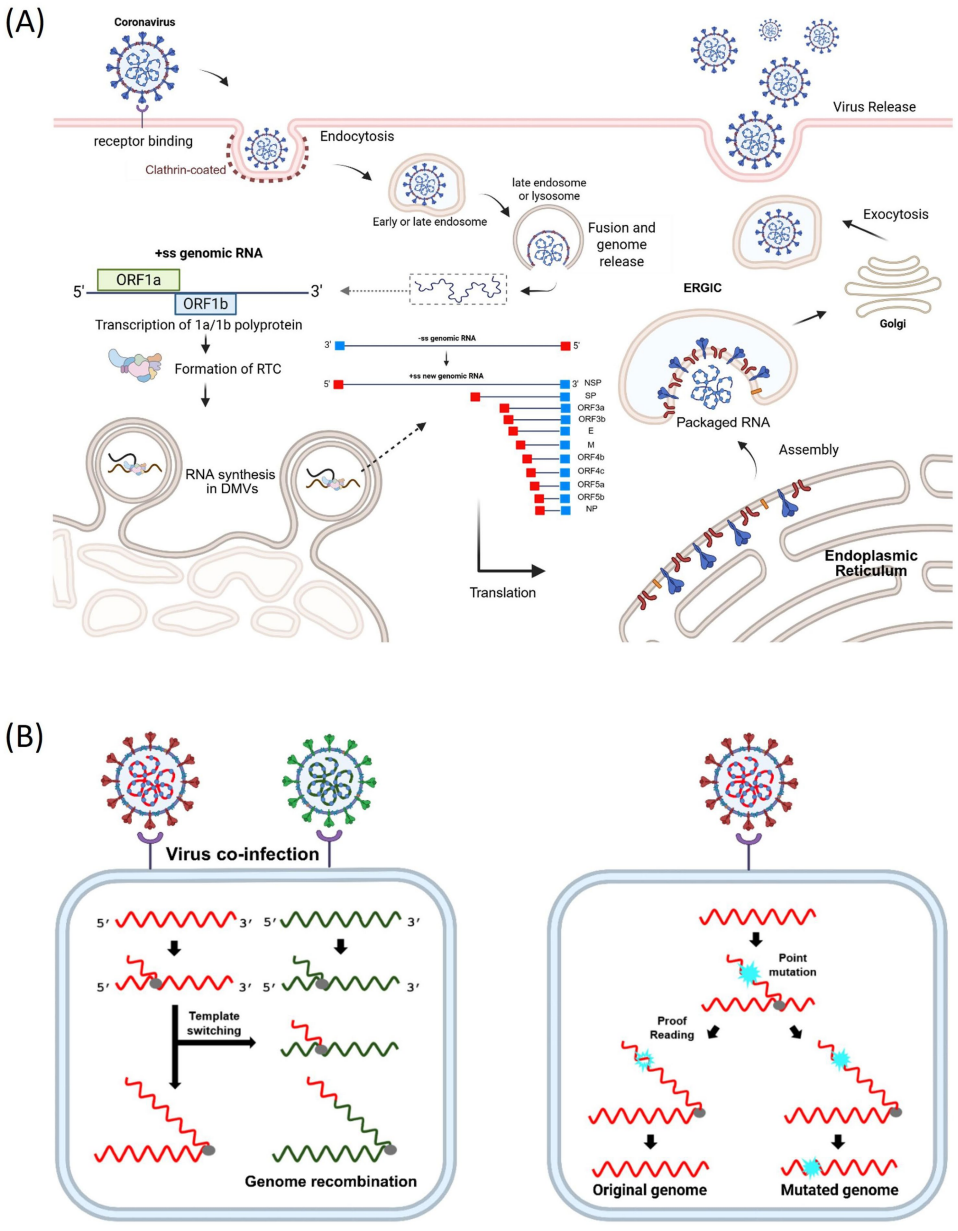
Coronavirus life cycle and mechanisms generating genetic diversity. **(A)** Schematic representation of the coronavirus life cycle, including viral attachment, entry, genome translation, replication–transcription complex formation, genome replication, virion assembly, and release. **(B)** Illustration of the mechanisms generating genetic diversity during coronavirus replication, including homologous recombination and point mutations introduced during RNA-dependent RNA polymerase–mediated genome replication.

The replication strategy of coronaviruses provides an important mechanistic basis for the remarkable evolutionary capacity of IBV. As schematically illustrated in Figure 1A, IBV replication, as in other coronaviruses, is mediated through a complex replication–transcription system that produces both full-length genomic RNA and a nested set of subgenomic messenger RNAs responsible for the expression of structural and accessory proteins (8, 47, 48).

IBV attachment involves interactions between host cell receptors and lipid raft domains on the host cell membrane, and subsequent entry is dependent on endosomal acidification (49, 50). IBV is primarily internalized via clathrin-mediated endocytosis (51). Following internalization, IBV is subsequently trafficked through the endosomal pathway, where low-pH–dependent membrane fusion enables release of the viral genome into the cytoplasm (51). Following entry, the genomic RNA is translated into replicase polyproteins, which are subsequently processed into non-structural proteins that form replication–transcription complexes (RTCs) on modified intracellular membranes (7, 52, 53). Within these complexes, the viral RdRp and associated cofactors mediate both genome replication and the transcription of subgenomic RNAs (sgRNAs) (7, 52, 54).

Recombination in positive-sense single-stranded RNA viruses, including coronaviruses, is thought to occur through RdRp-mediated template switching during negative-strand synthesis, a process that represents the initial step of both full-length genome replication and subgenomic RNA transcription (Figure 1B) (55, 56). During this process, RdRp may transiently dissociate from the RNA template and subsequently reassociate either at the same or a different position on the same molecule, or alternatively, with a different RNA template. Such template-switching events generate a variety of RNA products, including sgRNAs encoding structural and accessory proteins, defective RNAs (dRNAs), full-length genomic RNA, and recombinant genomes (56).

As a consequence of this recombination-prone and moderately error-prone replication strategy, recombination occurs frequently during coronavirus replication. The viral RNA polymerase complex can switch between homologous genomic templates during RNA synthesis, particularly when different IBV genotypes or lineages co-infect the same host cell (56). This process enables the incorporation of genetic fragments derived from distinct parental viruses into newly synthesized genomes, generating mosaic viral genomes with novel genetic combinations (57). Consistent with this mechanism, quantitative genomic studies have reported statistically significant S1 recombination in 14 of 94 IBV isolates (14.89%) and recombinant signatures in 6 of 8 newly identified Korean K-IIe isolates among 46 strains collected between 2003 and 2024 (34, 58). In addition, a European whole-genome analysis identified 215 recombination events across 69 IBV genomes, further indicating that recombination can occur repeatedly across the IBV genome (59). These findings indicate that recombination is repeatedly detected in IBV field populations and represents a major mechanism driving the emergence of new variants where multiple viral lineages commonly co-circulate (60–63).

In addition to recombination, point mutations introduced during RNA replication further contribute to viral diversification and immune escape in RNA viruses (Figure 1B) (64, 65). Although coronaviruses possess proofreading activity that partially reduces replication errors, mutation rates remain sufficiently high to generate substantial genetic variation over time (66–68). In IBV, reported evolutionary rates vary according to the genomic region, lineage, geographic scale, and analytical framework: a whole-genome-based analysis estimated a rate of approximately 10^−5^ substitutions/site/year, whereas S1-based analyses of the GI-19 lineage estimated higher rates of 2.71 × 10^−3^ and 3.22 × 10^−3^ substitutions/site/year in international and Italian datasets, respectively (19, 23). Consistent with this variation, comparative analyses of IBV structural genes have shown that the S1 region generally exhibits the highest evolutionary rate and genetic variability (23, 69), likely because mutations in this region can affect receptor binding, tissue tropism, antigenicity, and immune recognition (70–72). Under selective pressures imposed by host immunity and vaccination, variants carrying advantageous mutations may gradually expand within poultry populations (33, 73).

Collectively, the replication strategy of IBV integrates multiple mechanisms that promote rapid viral evolution, including discontinuous transcription, frequent template switching, and the continuous accumulation of point mutations. These processes generate genetically diverse viral populations capable of adapting to changing host environments and immune pressures. As a result, IBV populations in commercial poultry systems exhibit ongoing diversification and the periodic emergence of antigenically distinct variants (72, 74, 75). Understanding these replication-driven evolutionary processes provides a critical mechanistic framework for interpreting the molecular epidemiology, genotype turnover, and vaccine escape phenomena discussed in subsequent sections of this review.

## 3 Global genetic diversity and evolutionary dynamics of IBV

The S glycoprotein, particularly its S1 subunit, plays a central role in IBV evolution. As the primary determinant of host receptor binding and the major target of neutralizing antibodies, the S1 region accumulates mutations and recombination events that give rise to antigenically distinct variants (10, 33, 72). Consequently, phylogenetic classification based on the complete S1 gene has been widely adopted, leading to the identification of multiple genotypes (GI–GX) and lineages that reflect distinct evolutionary trajectories (14, 16, 25, 76–80). A notable feature of IBV epidemiology is the heterogeneity in the geographic distribution of lineages. While many variants remain geographically restricted, others have achieved global spread (15, 18). Historically, the Massachusetts (GI-1) and 793B (GI-13) lineages have been widely disseminated worldwide, largely influenced by the extensive use of live attenuated vaccines derived from these strains (15, 18, 25). In contrast, lineages such as GI-15 (Korea), GI-18 (Japan), and GI-26 (Africa) are confined to specific regions, reflecting local ecological and epidemiological conditions (43, 81, 82). GI-16 is estimated to have originated in either Europe or Asia and has subsequently been detected across multiple continents, including the Americas, Europe, Africa, and Asia (83, 84). GI-19, first identified in East Asia, has spread to major poultry-producing regions, including Europe and the Middle East, and remains a dominant lineage that has substantially shaped the global distribution of IBV over the past three decades (19, 21). Likewise, GI-23 originated in the Middle East and has expanded into Africa, Asia, and Europe, with its global significance increasing in recent years (20). When viewed collectively, the existence of lineages distributed across multiple continents suggests that IBV is capable of overcoming geographic barriers and spreading widely when favorable conditions are present.

According to recent analyses of the global evolutionary and dissemination patterns of the widely circulating GI-16, GI-19, and GI-23 lineages, the emergence of newly prevalent IBV variants does not necessarily reflect the *de novo* generation of new viruses (85). Rather, these lineages likely originated decades earlier, persisted at low levels within poultry populations, and subsequently expanded when favorable ecological or immunological conditions arose (21, 86). This interpretation is further supported by the evolutionary history of GI-7, which is currently prevalent in China and is estimated to have been introduced decades before its widespread detection (87). Thus, IBV evolution may be interpreted not only as a process of continual generation of novel variants, but also as one of latent lineage activation, in which pre-existing viral populations gain epidemiological advantage by exploiting ecological opportunities, such as introduction into immunologically naïve host populations, changes in vaccination strategies, or other shifts in poultry production systems. However, this framework does not exclude the possibility that newly generated variants can also spread rapidly. For example, recombinant variants derived from GI-13 and GI-19 lineages became prevalent in Italy and Spain (88), and the GI-17 DMV/1639/11-like strain showed a marked increase in prevalence after its first detection in Canada in 2015 (89). These examples indicate that recently generated recombinant variants, or newly recognized genotypes or lineages, can also acquire epidemiological advantage within a relatively short period. Therefore, the emergence and dissemination of IBV variants should be understood as the outcome of both the reactivation or expansion of pre-existing low-prevalence lineages and the rapid spread of newly generated variants arising through recombination or mutation.

Beyond the evolutionary processes that generate or favor particular IBV variants, their persistence and spread depend on the transmission pathways and ecological opportunities that allow them to circulate within and move between poultry populations. IBV transmission appears to occur primarily through sustained circulation within countries and short-distance spread between neighboring regions, whereas global dissemination occurs only intermittently through relatively rare introduction events (85). Consistent with this pattern, phylodynamic analyses have shown a positive correlation between poultry farm density and the rate of IBV spread, suggesting that densely concentrated poultry production areas may facilitate regional dissemination by increasing opportunities for direct or indirect transmission between farms (90, 91). In contrast, poultry trade has been considered one of the possible routes involved in the long-distance movement and interregional introduction of IBV (86). However, recent phylodynamic analyses suggest that the evidence is limited for considering trade as a consistent major driver of spread across all IBV lineages, and that its influence may vary depending on the lineage and epidemiological context (85). Therefore, IBV dissemination may not be limited to official animal movement networks, but may also occur through various indirect pathways, including human movement, contaminated equipment, vehicles, and contacts between farms (3, 15, 85, 90). In addition, in the case of migratory birds, their potential contribution to IBV spread has been proposed based on reports of coronavirus infection and the detection of IBV genetic material in wild birds (92–94). However, owing to the low detection frequency and limited available data, their role and epidemiological significance require further investigation (85, 95, 96).

Taken together, IBV evolution and dissemination are shaped by the interaction between viral genetic diversification and the ecological conditions that determine transmission. Variation in the S1 region generates antigenically and phylogenetically distinct lineages, but the emergence of prevalent variants cannot be explained solely by the continual generation of new viruses. Rather, global IBV patterns suggest that both the expansion of pre-existing low-prevalence lineages and the rapid spread of newly generated variants can occur when favorable ecological or immunological conditions arise. At the same time, lineage persistence and spread are strongly influenced by regional farm density, local transmission opportunities, trade-related movements, indirect transmission pathways, and potentially wild-bird interfaces. Therefore, IBV epidemiology should be understood as the outcome of dynamic interactions among viral evolution, host population connectivity, poultry production systems, and disease-control practices.

## 4 Poultry production systems in South Korea as drivers of IBV epidemiology and evolution

South Korea represents one of the most densely structured poultry production systems in East Asia, providing a unique ecological setting in which IBV transmission and evolution are strongly shaped by host population dynamics and production practices. Despite its relatively small geographic area, South Korea sustains a large and highly industrialized poultry sector. In 2025, Korea had approximately 96 million meat-type chickens and 79 million laying hens, with daily egg production reaching nearly 50 million eggs (29, 97). At the farm level, poultry production is characterized by large flock sizes and high stocking density. The average broiler farm in South Korea houses approximately 60,000 birds, although large integrated operations often exceed 100,000 birds per cycle, while individual layer farms typically house around 70,000 hens (29, 98). The concentration of such a large poultry population within a limited geographic space results in exceptionally high farm density, creating conditions that favor sustained viral transmission, mutation and persistence (99).

Beyond individual farms, the organizational structure of the poultry industry plays a critical role in shaping transmission dynamics. Vertically integrated production systems are widely implemented in broiler and native chicken sectors, linking breeding farms, hatcheries, feed mills, grow-out operations, and processing facilities into tightly connected networks (26, 27). The routine movement of day-old chicks, feed delivery vehicles, equipment, and farm personnel generates extensive epidemiological connectivity among farms. Even in the layer sector, where vertical integration is less pronounced, large-scale commercial farms are often geographically clustered, further enhancing regional pathogen transmission (91, 100, 101). Collectively, these features create a highly interconnected production network in which IBV can spread efficiently both within and between farms. From an epidemiological perspective, such a system provides ideal conditions for local persistence and regional circulation, which are recognized as dominant drivers of IBV spread.

Taken as a whole, the poultry production system in South Korea functions as an integrated ecological platform that drives both IBV transmission and evolution. High host density, large flock sizes, and strong network connectivity sustain viral circulation. These interacting factors create a dynamic environment in which IBV populations continuously adapt, diversify, and reorganize over time. From this perspective, IBV epidemiology in South Korea cannot be understood solely in terms of viral properties, but must be interpreted within the broader context of production system structure and management practices. The Korean poultry industry therefore provides a valuable real-world model demonstrating how host population dynamics and transmission networks collectively shape the evolutionary behavior of rapidly adapting RNA viruses.

## 5 Intra-lineage diversification and population structuring of IBV in Korea

**Figure 2:**
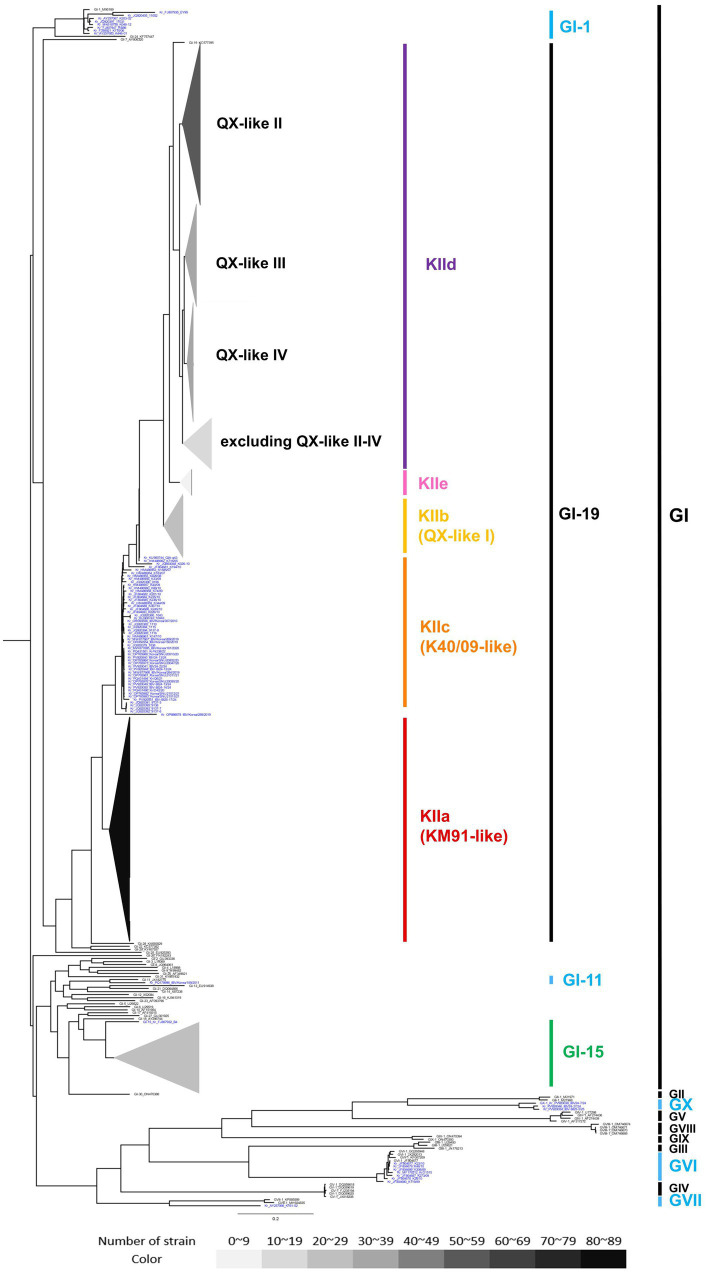
Phylogenetic analysis of global infectious bronchitis virus (IBV) genotypes and Korean strains based on S1 gene sequences. The phylogenetic tree was constructed using representative IBV strains worldwide, together with Korean strains for which full-length S1 gene sequences were available in GenBank. Phylogenetic analysis was performed using the maximum likelihood method implemented in IQ-TREE, with bootstrap analysis (1,000 replicates). Tree visualization was carried out using FigTree v1.4.4. Korean IBV sublineages within major GI lineages are indicated by triangles, while GI-1 and GI-11 were not specifically highlighted due to their limited representation. The GI-19 sublineage KIIc, identified as a recombinant lineage, was not grouped into a single clade and therefore was not represented by a triangle. The remaining GI lineages are represented using a grayscale gradient (light gray to black) to reflect relative population size. GI-19 sublineages are distinguished by color: KIIa (red), KIIb (yellow), KIIc (orange), KIId (purple), and KIIe(pink), while GI-15 is shown in green. Less frequently reported genotypes and lineages (GI-1, GI-11, GVI, GVII, GX) are indicated in sky blue. Korean strains are highlighted in blue taxa. Detailed information of the reference strains and Korean strains are provided in Supplementary Tables S1, S2.

The long-term molecular epidemiology of IBV in South Korea provides a unique framework for understanding how rapidly evolving RNA viruses adapt within intensive poultry production systems. Rather than exhibiting genetic stability or simple linear replacement, IBV populations in Korea have undergone continuous restructuring over several decades, characterized by repeated lineage emergence, diversification, and replacement (16, 32–43). Phylogenetic analysis based on the S1 gene demonstrates that Korean IBV strains are distributed across multiple globally recognized genotypes and lineages, reflecting a complex evolutionary history shaped by repeated viral introduction and local diversification (Figure 2). In particular, this phylogenetic structure does not primarily represent the emergence of entirely new viruses, but rather the expansion of pre-existing viral lineages.

Historically, IBV was first isolated in South Korea in 1986 as a Massachusetts-type virus (GI-1 lineage), although serological evidence indicates that the virus had likely circulated prior to its detection (102). A major epidemiological transition occurred in the early 1990s with the emergence of nephropathogenic strains, most notably KM91-like viruses belonging to the GI-19 lineage (35, 103). These viruses rapidly became dominant due to their enhanced pathogenicity and transmission efficiency, fundamentally reshaping the clinical and economic landscape of IB in Korea (35, 37, 39). Subsequent evolutionary dynamics were not characterized by simple lineage replacement but by increasing population complexity driven by the long-term co-circulation of multiple IBV lineages. Variants belonging to GI-19, GI-15, and recombinant clusters have co-existed within poultry populations, forming a structured viral community in which distinct lineages expand, compete, and decline over time (33, 43).

The GI-15 lineage identified in Korea during the 2000s was designated as the KI group, whereas the GI-19 lineage was classified as the KII group (39). Among these, the GI-19 lineage underwent rapid compression and recombination within the Korean poultry industry, which is characterized by specific vaccine pressure, high-density farming conditions, large-scale poultry production systems, and extensive inter-farm connectivity. As a result, by 2025, the GI-19 lineage had diversified into five sublineages, designated KIIa–KIIe (34). Notably, the KIId sublineage was further subdivided into at least three distinct subgroups, namely QXII–QXIV (33, 43, 104). Interestingly, these sublineages did not coexist simultaneously at equal prevalence, but rather exhibited sequential dominance patterns depending on the period (33). This pattern is consistent with the characteristic temporal shifts in predominant IBV lineages and reflects the continuous genetic diversification of viral populations (5, 15, 18). Such dynamics may be interpreted as the result of lineage competition, in which viral lineages co-circulating within a shared host population compete under immune selection pressure, thereby allowing certain lineages to preferentially expand (85). In addition, frequent recombination events occurred among the co-circulating IBV genotypes (40, 104–110). A representative example is the emergence of the novel GI-19 sublineage, KIIc, through recombination between the KIIa and KIIb lineages. These recombination events are considered to play a critical role in accelerating the genetic diversity and evolutionary dynamics of IBV (40).

These evolutionary trajectories of the GI-19 lineage have been reported not only in China but also in other countries (15, 19, 34). In China, although multiple genotypes and lineages have been co-circulating simultaneously, the GI-19 lineage is recognized as having originated there and has continued to exhibit a higher prevalence compared with other genotypes and lineages (46, 80, 111, 112). Within this context, the genetic diversity of the S1 gene in the GI-19 lineage has been identified (113). In particular, whole-genome phylogenetic and Simplot analyses demonstrated the presence of genetic differences between viral populations before and after 2006 (24). This phenomenon was presumed to result from the temporary accumulation and persistence of IBV mutations under vaccine-induced selective pressure, while viruses identified after 2006 may have acquired greater fitness for replication *in vivo* (114). Furthermore, a study by Xu et al. in 2018 demonstrated that the GI-19 lineage circulating in China could be further classified into seven sublineages (A–G), and the predominance of specific sublineages was also reported (115). Moreover, a recent study on the GI-19 lineage reported in Spain demonstrated the presence of three major clades and several minor clades based on partial S1 sequence analysis, and vaccine-derived recombinant strains between the GI-13 lineage and the GI-19 lineage were also identified (116). In addition, a recent study investigating the global spread and epidemiology of the GI-19 lineage reported that, following a single introduction event, the GI-19 lineage evolved independently within each continent and country (21). These findings suggest that, in countries and regions where the GI-19 lineage remains endemic, similar to the situation in Korea, the lineage has been continuously interacting with vaccine strains and exposed to various selective pressures, thereby acquiring region-specific genetic diversity through independent evolutionary processes (33, 34, 43).

The continuous genetic diversification of the GI-19 lineage is not limited to phylogenetic divergence alone, but is also closely associated with differences in tissue tropism and pathogenicity among genotypes. In particular, in Korea, the nephropathogenic GI-19 lineage has been associated with severe renal lesions and high mortality, whereas other lineages, such as GI-15, primarily induce respiratory disease and exhibit relatively lower pathogenicity (16, 35, 110). These differences have important implications for the establishment of national vaccine development and disease control strategies, and provide the basis for the emphasis on the development of vaccines targeting the GI-19 lineage in Korea (117–120). Therefore, the ongoing evolution and genetic diversification of the GI-19 lineage are considered to play an important role in shaping the broad clinical manifestations of IBV infections observed in Korea.

Taken together, the phylogenetic structure observed in South Korea represents the outcome of an integrated evolutionary system (Figure 2), in which viral genetic mechanisms, host population dynamics, and vaccination practices interact to shape viral population trajectories. IBV epidemiology can therefore be understood as a self-reinforcing evolutionary system, characterized by continuous diversification, lineage competition, and periodic replacement of dominant variants. This integrated framework highlights that IBV evolution is not driven by vaccination alone but emerges from the combined effects of host density, local transmission dynamics, viral genetic plasticity, and immune selection. The Korean IBV system thus provides a valuable longitudinal model for understanding the evolutionary behavior of rapidly adapting RNA viruses under sustained ecological and immunological pressures, with broader implications for the control of emerging coronaviruses.

## 6 Vaccination strategies and practical challenges in IBV control

Vaccination constitutes the cornerstone of IBV control in South Korea. Over time, vaccines have been continuously developed and introduced in response to the evolving IBV landscape, and the approval timelines for vaccines targeting specific lineages and sublineages are summarized in Table 1 (117–120). In practice, however, its implementation reflects not only immunological considerations but also the operational realities of modern poultry production systems. Consequently, vaccination strategies are highly structured and standardized across production stages, forming an integral component of disease management in both broiler and layer sectors (121, 122).

**Table 1:** IBV vaccine strains approved by regulatory authorities in South Korea.

Year^a^	Strain	Genotype (sublineage)^b^	Vaccine type
1986	H-120(Mass)	GI-1	Live attenuated
1987	M41(Mass)	GI-1	Inactivated
1993	1,263	GI-1	Live attenuated
1996	KM91	GI-19 (KIIa)	Inactivated
1998	Ma5(Mass)	GI-1	Live attenuated
2009	K2	GI-19 (KIIa)	Live attenuated
2012	ADL05258	G1-19 (ND^b^)	Inactivated
2014	AVN2/08	GI-19 (KIIb)	Inactivated
2015	AVR1/08	GI-15	Live attenuated
2015	K40/09	GI-19 (KIIc)	Inactivated
2018	CakII	GI-19 (KIIa)	Live attenuated
2018	K40/09	GI-19 (KIIc)	Live attenuated
2021	KM-QXE10	G1-19 (ND)	Inactivated
2021	KM-QXE120	G1-19 (ND)	Live attenuated
2023	K1277/03 P90	GI-19 (KIIb)	Live attenuated
2023	CV001 CV013	GI-19 (KIId) GI-15	Inactivated
2024	QX 1830029	GI-19 (KIId)	Live attenuated

a The year indicates when each strain was first approved as a live or inactivated vaccine strain in South Korea.

b Sublineage not determined.

In commercial hatcheries, live attenuated vaccines are routinely administered at day-old, most commonly via spray application (121, 122). This approach ensures uniform vaccine coverage across large populations and contributes to the development of early mucosal immunity in highly susceptible chicks. At the farm level, additional live attenuated IBV vaccines are typically administered between 10 and 20 days of age, primarily via spray application or drinking water (121, 122). This method enables rapid mass vaccination and reinforces immunity during the early production phase, when birds are increasingly exposed to environmental pathogens. Together, hatchery spray vaccination and farm-level drinking water administration represent the primary strategy for early-life immunization in Korean poultry systems. For long-lived flocks, including layers and breeders, inactivated vaccines are used as booster immunizations following live vaccination (121, 122). These vaccines are commonly formulated as multivalent oil-emulsion preparations, incorporating IBV together with other economically important pathogens and such multivalent vaccines are typically administered 3–4 weeks prior to the onset of lay to enhance and sustain systemic immunity throughout the production period. This strategy reflects both immunological requirements and practical feasibility, as individual injection is largely restricted to long-lived birds.

**Figure 3:**
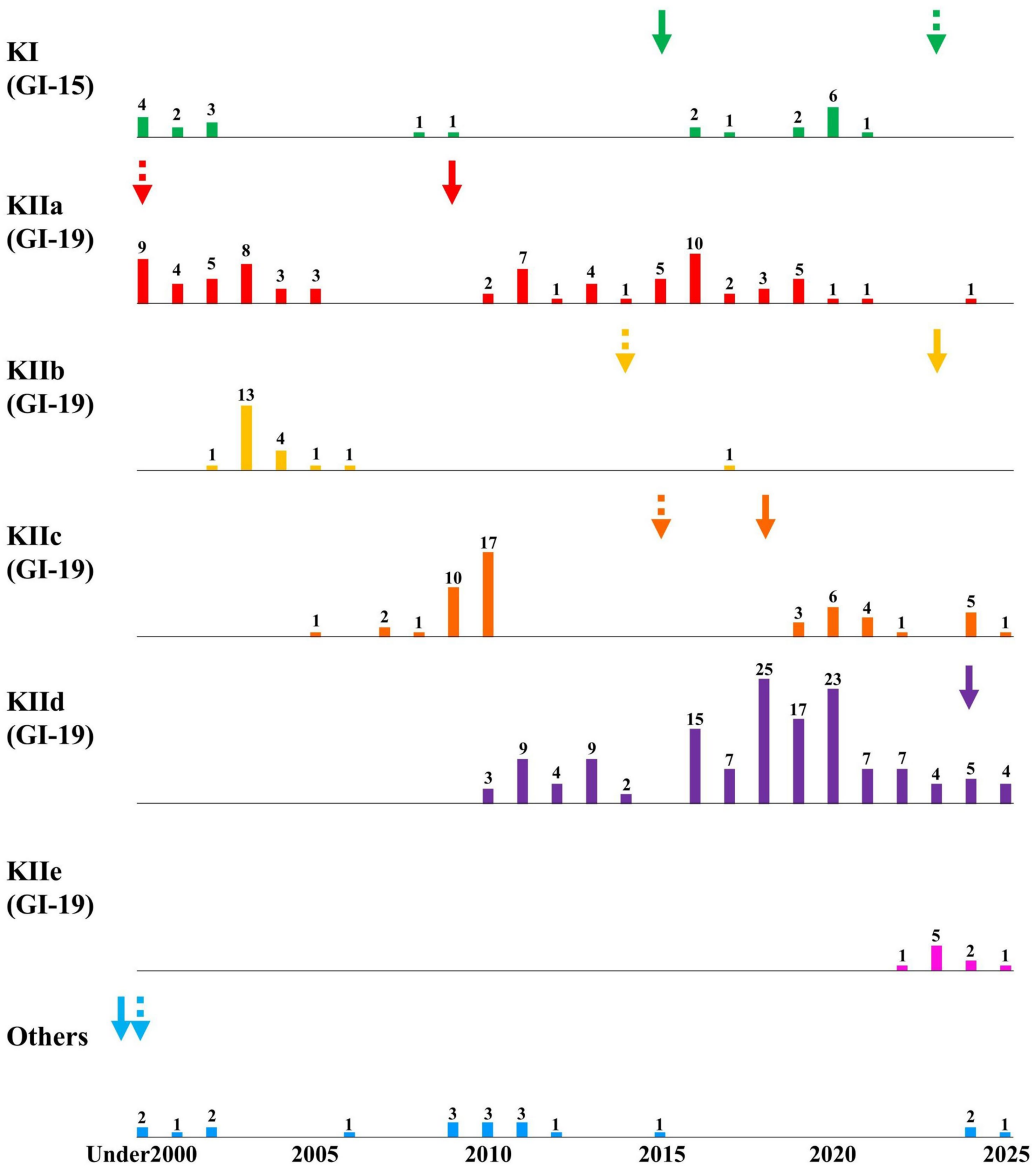
Temporal distribution of infectious bronchitis virus (IBV) genotypes circulating in South Korea and timeline of vaccine introduction. The distribution of major genotypes, including GI-19 sublineages (KIIa–KIIe) and GI-15, is presented over time. Genotypes sporadically detected in Korea (GI-1, GI-11, GVI-1, GVII-1, GX-1) are grouped as “Others.” The timing of initial approval for vaccines corresponding to each lineage or sublineage is indicated by arrows. Solid arrows represent live attenuated vaccines, while dashed arrows indicate inactivated vaccines. GI-19 sublineage are distinguished by color: KIIa (red), KIIb (yellow), KIIc (orange), KIId (purple), and KIIe (pink), while GI-15 is shown in green. “Others” are indicated in sky blue.

The evolution of these vaccination strategies has closely paralleled the changing epidemiology of IBV in South Korea (Figure 3). As new variants have emerged and replaced previously dominant lineages, vaccination programs have been repeatedly adjusted through the introduction of new vaccine strains and combinations (117–120). However, this adaptive process has not fully compensated for the rapid evolutionary dynamics of IBV, because the turnover of viral lineages often outpaces the rate at which vaccine strains can be updated. This temporal lag may result in recurrent antigenic mismatch, thereby contributing to the continuous reshaping of viral evolutionary trajectories (5, 8, 33). These limitations are evident in current vaccination strategies, and as illustrated in Figure 3, a temporal mismatch between viral evolution and vaccine updates has also been observed in South Korea. Notably, the emergence and dominance of major IBV sublineages frequently precede the introduction or widespread use of corresponding vaccine strains. This lag can be attributed to the time required for surveillance, characterization of circulating strains, vaccine strain selection, vaccine development, and regulatory approval. Consequently, antigenic divergence between circulating viruses and vaccine strains arises, leading to reduced cross-protective immunity and, in some cases, decreased vaccine efficacy or vaccination failure (5, 123).

These observations indicate that future IBV vaccination strategies should be designed not only to improve protective efficacy, but also to account for their potential effects on viral evolutionary dynamics. From a practical perspective, future IBV vaccination strategies must be considered within the logistical constraints of intensive poultry production systems. Live attenuated vaccines remain widely used not only because they are inexpensive, easy to administer, and suitable for mass application, but also because they can induce local mucosal immunity in the respiratory tract, thereby reducing early viral replication and viral shedding following infection (122). However, despite these advantages, live attenuated vaccines also possess inherent limitations, including the potential risk of recombination with circulating field strains (63, 124, 125). Accordingly, future research and development efforts should focus on complementary vaccine platforms capable of preserving or enhancing the mucosal immune protection conferred by live attenuated vaccines while simultaneously improving biosafety. In this context, emerging vaccine modalities and advanced delivery technologies may provide promising alternatives. Recombinant vector-based vaccines, subunit vaccines, DNA vaccines, and mRNA vaccines may contribute to this goal, particularly if they are optimized to induce mucosal immunity (126–129). In addition, subunit or inactivated antigen formulations combined with mucosal adjuvants, nanoparticle-based delivery systems, or oculo-nasal administration may further promote local IgA responses at the respiratory mucosa (130). Importantly, the development of such next-generation vaccine platforms must also consider the practical realities of the poultry industry, particularly the requirement for cost-effective mass vaccination (122). Therefore, future IBV vaccine strategies should aim not only to achieve robust mucosal immunity and improved safety profiles, but also to ensure economic feasibility, scalability, and field applicability within high-density poultry production systems.

In summary, vaccination in South Korea should be understood not only as a disease control measure, but also as a central component of the IBV evolutionary system. The interaction between high host density, continuous viral circulation, repeated vaccination, and delayed vaccine updates creates conditions that favor immune selection, recombination, and lineage competition. Within this dynamic framework, IBV evolution is continuously shaped by the balance between vaccine-induced immunity and viral adaptation. Therefore, effective long-term control of IBV will require an integrated strategy combining optimized vaccination programs, continuous molecular surveillance, and a deeper understanding of vaccine-driven viral evolution. In particular, broad cross-lineage protection, timely vaccine strain updates, and enhanced mucosal immunity at the respiratory entry site will be critical for reducing viral replication, shedding, and the evolutionary pressures that sustain viral diversification. While live attenuated vaccines will likely remain indispensable in the near term, future vaccine development should explore complementary platforms that can provide broader and more durable protection without increasing reliance on conventional live vaccine strategies. This integrated approach may help limit transmission, recombination opportunities, and immune escape in high-density poultry systems.

## 7 Vaccination-driven viral evolution: implications for control of coronavirus infections

The Korean experience provides a compelling conceptual framework for understanding IBV control within highly dynamic production systems, in which vaccination plays a central yet inherently paradoxical role. In intensive poultry environments, IBV does not persist as a single dominant strain but as a continuously shifting population composed of multiple co-circulating lineages. This population structure, sustained by high host density and extensive connectivity, creates an ecological landscape that inherently favors viral diversification and adaptation (5, 15, 18, 85).

**Figure 4:**
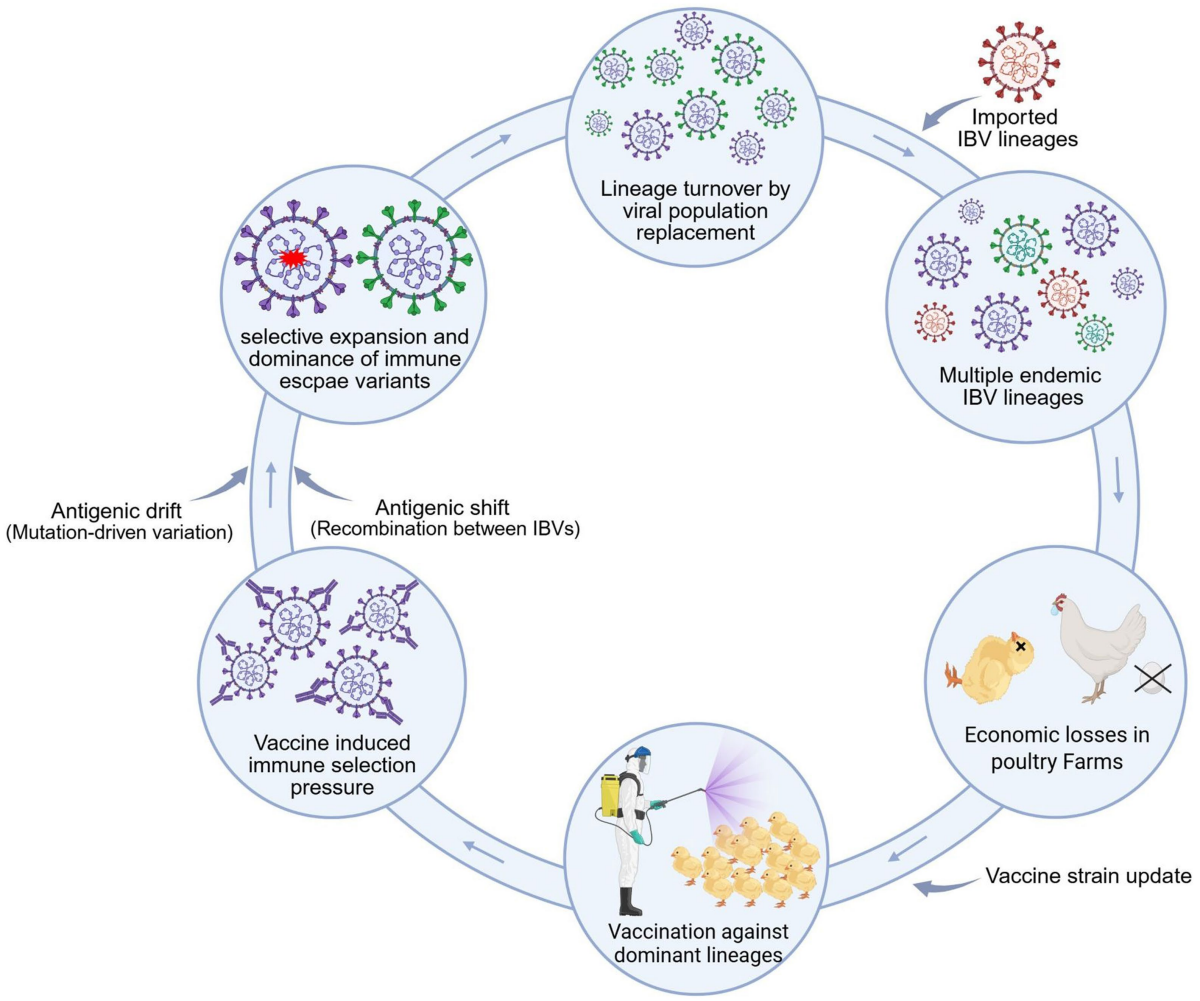
Cyclical dynamics of vaccination-driven evolution in infectious bronchitis virus (IBV). This conceptual model illustrates a self-perpetuating evolutionary cycle in which the introduction of novel lineages, together with vaccine-imposed immune selection and recombination among co-circulating field and vaccine-derived strains, drives the emergence and expansion of antigenically distinct variants. As these variants spread, they progressively reduce the effectiveness of existing vaccines, leading to antigenic mismatch and necessitating the development and deployment of updated vaccines. However, each vaccination intervention reshapes the selective landscape, generating new immune pressures that favor the emergence of additional variants. This iterative cycle—comprising variant emergence, immune selection, vaccine update, and renewed diversification—continuously fuels viral diversity and lineage turnover. Collectively, these processes highlight vaccination not only as a control measure but also as a central force sustaining the evolutionary dynamics of IBV.

Within this framework, vaccination should be understood not merely as a protective intervention, but as one of the key ecological forces that actively restructure viral population dynamics (Figure 4). Under conditions of continuous viral circulation, vaccination imposes sustained immune selection pressure, reshaping the relative fitness of circulating variants and, consequently, altering lineage dominance patterns (33, 73). Furthermore, the use of live attenuated vaccines expands the viral gene pool and promotes the co-circulation of vaccine-derived and field strains, thereby increasing the likelihood of co-infection and genetic exchange (63, 91, 124). Under such conditions, immune selection drives the gradual accumulation of antigenic changes, while recombination can generate mosaic genomes, ultimately accelerating viral diversification.

These interacting processes give rise to a characteristic evolutionary trajectory: the emergence of antigenically distinct variants, their selective expansion under immune pressure, and the eventual replacement of previously dominant lineages (5, 15, 122). As antigenic divergence accumulates between circulating viruses and vaccine strains, antigenic mismatch inevitably develops, leading to reduced vaccine efficacy and, in some cases, vaccination failure. Although periodic updates of vaccine strains are required to restore protection, such interventions re-establish selective pressure, thereby perpetuating a self-sustaining cycle of viral diversification and immune escape.

Notably, this vaccination-driven evolutionary cycle is not unique to IBV but reflects generalizable principles applicable across RNA viruses. Similar dynamics are well documented in influenza viruses, where population-level immunity and vaccination drive antigenic drift and lineage turnover. Although the mechanisms of genetic exchange differ between influenza viruses and coronaviruses—reassortment in influenza viruses versus recombination in IBV—the co-circulation of multiple viral populations similarly facilitates viral diversification (131–134). These parallels highlight that vaccination environments can function as powerful evolutionary systems, shaping both the direction and rate of viral evolution.

Moreover, these principles may extend beyond animal viruses to human pathogens. During the SARS-CoV-2 pandemic, widespread vaccination and the increase in population-level immunity were closely associated with repeated patterns of variant emergence, dissemination, and replacement (65, 135–137). These phenomena share certain similarities with the immune selection pressure-driven evolutionary patterns observed in the IBV system, suggesting that the long-term evolution of emerging coronaviruses may likewise follow comparable trajectories under sustained immune pressure. However, important ecological and epidemiological differences exist between the two viral systems. Whereas SARS-CoV-2 variant dynamics have largely been shaped in a globally connected human population in which infection- and recovery-induced immunity, together with Spike-focused vaccine-induced immunity, accumulated rapidly, the IBV scenario discussed in this study is characterized by sustained immune pressure imposed by long-term repeated use of live vaccines, gradual antigenic drift, and frequent recombination as major drivers of viral diversification (73, 122, 124, 138, 139).

From this perspective, the IBV system provides a forward-looking model for anticipating evolutionary risk. In environments characterized by high transmission intensity, prolonged co-circulation of multiple lineages, and continuous immune selection, viral populations are likely to exhibit increased genetic diversity, frequent recombination, and accelerated emergence of variants with enhanced transmissibility or immune escape capacity. These features represent critical early warning signals for the evolution of high-risk viral phenotypes. Accordingly, effective control strategies must move beyond static vaccination approaches and instead adopt an integrated, evolution-aware framework. Key priorities include reducing opportunities for co-circulation and mixed infection, strengthening real-time molecular surveillance, accelerating the updating of vaccine strains, and developing vaccination strategies that balance protective immunity with the minimization of selective pressure (33, 73).

Within a One Health context, IBV represents a uniquely informative natural model of vaccine-driven viral evolution observed over multiple decades in real-world production systems. Unlike human outbreaks, which are often constrained by shorter observational timescales focused on immediate transmission dynamics (140, 141), the IBV system provides longitudinal evidence demonstrating how host population structure, viral ecology, and vaccination practices interact to shape evolutionary outcomes.

The conceptual model presented in Figure 4 reveals a fundamental paradigm shift: vaccination should be understood not only as a tool for disease control, but also as an important selective force among multiple interacting factors of viral evolutionary trajectories. Recognizing and integrating this dual role will be essential for developing sustainable strategies to control rapidly evolving RNA viruses in both animal and human populations.

## 8 Conclusion

IBV remains one of the most important viral pathogens in modern poultry production because of its extensive genetic diversity and continuous capacity for adaptive evolution. Long-term epidemiological studies of IBV in Korea provide a useful real-world model for understanding how viral populations evolve under conditions of high host density, sustained viral transmission, and intensive vaccination. Within such production systems, the persistent coexistence and circulation of multiple lineages, frequent recombination, lineage introduction, and immune selection create a highly dynamic viral ecosystem. A key concept emerging from this study is a vaccination-driven evolutionary cycle. High host density, extensive inter-farm connectivity, and the continued use of live attenuated vaccines may further create conditions favorable for viral persistence, co-infection, and genetic exchange, thereby accelerating viral evolution. Therefore, vaccination should be understood not merely as a disease-prevention measure, but also one of the potential ecological and evolutionary factors shaping the evolutionary trajectory of IBV.

Importantly, the evolutionary principles observed in IBV may offer broader insights into rapidly evolving RNA viruses beyond avian systems. In particular, the repeated emergence, spread, and replacement of immune-escape variants during the SARS-CoV-2 pandemic illustrate broadly comparable evolutionary pressures acting under widespread population immunity. In this respect, the IBV system provides a useful comparative model for understanding the long-term evolutionary dynamics of coronaviruses and other RNA viruses under sustained immune pressure. Collectively, these findings suggest the need for a conceptual shift in viral disease control. Effective long-term control requires adaptive, evolution-informed strategies that integrate continuous molecular surveillance, real-time monitoring of viral population changes, and flexible vaccine strategies capable of rapidly responding to antigenic change. Ultimately, the sustainable management of IBV and other rapidly evolving RNA viruses depends on our ability to predict how viruses change and spread in the field and to manage these processes within real-world production systems.
