# Supplementary material for: Vaccination-driven evolution of infectious bronchitis virus in Korea: implication for the control of other coronavirus infections
Source: Front Vet Sci. 2026 Jun 23;13:1859772. doi: 10.3389/fvets.2026.1859772 (PMC13337371; doi:10.3389/fvets.2026.1859772)
Supplement: Supplementary file 1 [file Table_1.DOCX]

**Supplementary Table S1. List of reference strains and sequence information used for global IBV genotype phylogenetic analysis**

| Strain | Lineage | Country | Isolated Year | Accession number |
| --- | --- | --- | --- | --- |
| Beaudette | GI-1 | USA | 1937 | M95169 |
| Holte | GI-2 | USA | 1954 | GU393336 |
| Gray | GI-3 | USA | 1960 | L14069 |
| Holte | GI-4 | USA | 1962 | L18988 |
| N1/62 | GI-5 | Australia | 1962 | U29522 |
| VicS | GI-6 | Australia | 1962 | U29519 |
| TP/64 | GI-7 | Taiwan | 1964 | AY606320 |
| L718 | GI-8 | USA | 1966 | JQ964067 |
| ARK99 | GI-9 | USA | 1973 | M99482 |
| B | GI-10 | New Zealand | 1970s | AF151954 |
| IBV/Brasil/351/1984 | GI-11 | Brazil | 1984 | GU393339 |
| D3896 | GI-12 | The Netherlands | 1978 | X52084 |
| Moroccan-G/83 | GI-13 | Morocco | 1983 | EU914938 |
| B1648 | GI-14 | Belgium | 1984 | X87238 |
| B4 | GI-15 | Korea | 1986 | FJ807932 |
| IZO 28/86 | GI-16 | Italy | 1986 | KJ941019 |
| CA/Machado/88 | GI-17 | USA | 1988 | AF419315 |
| JP8127 | GI-18 | Japan | 1996 | AY296744 |
| 58Hen-93II | GI-19 | China | 1997 | KC577395 |
| Qu_mv | GI-20 | Canada | 1996 | AF349621 |
| Spain/97/314 | GI-21 | Spain | 1997 | DQ064806 |
| 40/GDGZ-97I | GI-22 | China | 1997 | KC577382 |
| Variant2 | GI-23 | Israel | 1998 | AF093796 |
| V13 | GI-24 | India | 1998 | KF757447 |
| CA/1737/04 | GI-25 | USA | 2004 | EU925393 |
| NGA/B401/2006 | GI-26 | Nigeria | 2006 | FN182243 |
| GA08 | GI-27 | USA | 2008 | GU301925 |
| ck/CH/LGX/111119 | GI-28 | China | 2011 | KX640829 |
| gammaCoV/ck/China/I0111/14 | GI-29 | China | 2014 | KY407557 |
| Mex-07-1 | GI-30 | Mexico | 2007 | ON470386 |
| D2334/11/2/13/CI | GI-31 | Cote d'Ivoire | 2013 | MZ325299 |
| D1466 | GII-1 | The Netherlands | 1979 | M21971 |
| N1/88 | GIII-1 | Australia | 1988 | U29450 |
| DE/072/92 | GIV-1 | USA | 1992 | U77298 |
| N4/02 | GV-1 | Australia | 2002 | DQ059618 |
| TC07-2 | GVI-1 | China | 2002 | GQ265948 |
| V1397 | GII-1 | The Netherlands | 1980s | M21968 |
| N1/08 | GIII-1 | Australia | 2008 | JN176213 |
| V18/91 | GIII-1 | Australia | 1991 | U29521 |
| GA/5381/99 | GIV-1 | USA | 1999 | AF274439 |
| CU82616 | GIV-1 | USA | NA | AF317212 |
| AR/6386/97 | GIV-1 | USA | 1997 | AF274436 |
| 018 | GV-1 | Australia | 2008 | JX018208 |
| NS/03 | GV-1 | Australia | 2003 | DQ059619 |
| N1/03 | GV-1 | Australia | 2003 | FJ235194 |
| N4/03 (S1 | GV-1 | Australia | 2003 | DQ059620 |
| K23/10 | GVI-1 | Korea | 2010 | JF804677 |
| SDIB781/2012 | GVI-1 | China | 2012 | KF007209 |
| GX-NN09032 | GVI-1 | China | 2009 | JX292013 |
| GX-NN130021 | GVII-1 | China | 2013 | KP085589 |
| ck/China/I0636/16 | GVII-1 | China | 2016 | MH924835 |
| Ck/DE/AC-36213/02/2021 | GVIII-1 | Germany | 2021 | OM746674 |
| Ck/DE/AC-04987/02/2016 | GVIII-1 | Germany | 2016 | OM746671 |
| Ck/DE/AC-04677/03/2016 | GVIII-1 | Germany | 2016 | OM746670 |
| Ck/DE/AC-01811/03/2016 | GVIII-1 | Germany | 2016 | OM746669 |
| ON470394 Mex-56-7 | GIX-1 | Mexico | 2007 | ON470394 |
| Mex-14P | GIX-1 | Mexico | 2020 | ON470393 |
| SNU24007 | GX-1 | Korea | 2024 | PV920038 |
| SNU24027 | GX-1 | Korea | 2024 | PV920046 |
| SNU-BI25-5 | GX-1 | Korea | 2025 | PV920054 |
