# Supplementary material for: Vaccination-driven evolution of infectious bronchitis virus in Korea: implication for the control of other coronavirus infections
Source: Front Vet Sci. 2026 Jun 23;13:1859772. doi: 10.3389/fvets.2026.1859772 (PMC13337371; doi:10.3389/fvets.2026.1859772)
Supplement: Supplementary file 2 [file Table_2.DOCX]

**Supplementary Table S2. Korean IBV reference strains and associated sequence information used for phylogenetic analysis**

| Strain | Genotype | GI-19 Subgenotype | Isolated year | Accession Number | Reference |
| --- | --- | --- | --- | --- | --- |
| B4 | GI-15 | - | 1986 | FJ807932 | (36) |
| EJ95 | GI-15 | - | 1995 | FJ807933 |  |
| EY95 | GI-1 | - | 1995 | FJ807935 |  |
| K348/99 | GI-15 | - | 1999 | FJ807940 |  |
| KC90 | GI-19 | KIIa | 1990 | FJ807945 |  |
| KM91 | GI-19 | KIIa | 1991 | FJ807946 |  |
| K151/98 | GI-19 | KIIa | 1998 | FJ807937 |  |
| K152/98 | GI-19 | KIIa | 1998 | FJ807938 |  |
| K083/98 | GI-19 | KIIa | 1998 | FJ807936 |  |
| K242/99 | GI-19 | KIIa | 1999 | FJ807939 |  |
| K451/99 | GI-19 | KIIa | 1999 | FJ807941 |  |
| K576/99 | GI-19 | KIIa | 1999 | FJ807943 |  |
| ES90 | GI-19 | KIIa | 1990 | FJ807934 |  |
| K571/99 | GI-15 | - | 1999 | FJ807942 |  |
| K620/02 | GI-15 | - | 2002 | FJ807944 |  |
| RB86 | GI-1 | - | 1986 | FJ807947 |  |
| K069-01 | GI-19 | KIIa | 2001 | AY257061 | (37) |
| K281-01 | GI-15 | - | 2001 | AY257062 |  |
| K434-01 | GI-15 | - | 2001 | AY790368 |  |
| K446-01 | GI-1 | - | 2001 | AY257063 |  |
| K507-01 | GI-19 | KIIa | 2001 | AY257064 |  |
| K748-01 | GI-19 | KIIa | 2001 | AY790358 |  |
| K774-01 | GI-19 | KIIa | 2001 | AY257065 |  |
| K044-02 | GI-19 | KIIa | 2002 | AY790365 |  |
| K058-02 | GI-19 | KIIa | 2002 | AY790361 |  |
| K117-02 | GI-19 | KIIa | 2002 | AY790359 |  |
| K142-02 | GI-19 | KIIa | 2002 | AY257060 |  |
| K161-02 | GVII-1 | - | 2002 | AY257066 |  |
| K203-02 | GI-1 | - | 2002 | AY257067 |  |
| K210-02 | GI-15 | - | 2002 | AY257068 |  |
| K234-02 | GI-19 | KIIa | 2002 | AY790362 |  |
| K545-02 | GI-15 | - | 2002 | AY790366 | (38) |
| K514-03 | GI-19 | KIIa | 2003 | AY790360 |  |
| K10217-03 | GI-19 | KIIb | 2003 | AY790363 |  |
| K1255-03 | GI-19 | KIIb | 2003 | AY790364 |  |
| K035/04 | GI-19 | KIIb | 2004 | FJ80920 | (39) |
| K110/06 | GI-1 | - | 2006 | FJ80921 |  |
| K154/05 | GI-19 | KIIb | 2005 | FJ80922 |  |
| K283/04 | GI-19 | KIIb | 2004 | FJ80923 |  |
| K463/04 | GI-19 | KIIb | 2004 | FJ80924 |  |
| K630/02 | GI-19 | KIIb | 2002 | FJ80925 |  |
| K961/04 | GI-19 | KIIa | 2004 | FJ80926 |  |
| K1019/03 | GI-19 | KIIb | 2003 | FJ80927 |  |
| K1255/03 | GI-19 | KIIb | 2003 | FJ80928 |  |
| K1257/03 | GI-19 | KIIb | 2003 | FJ80929 |  |
| K1277/03 | GI-19 | KIIb | 2003 | FJ80930 |  |
| K1583/04 | GI-19 | KIIb | 2004 | FJ80931 |  |
| SNU8067 | GI-15 | - | 2008 | JQ977697 | (41) |
| K1585/07 | GI-19 | KIIc | 2007 | HM486953 | (40) |
| K183/07 | GI-19 | KIIc | 2007 | HM486954 |  |
| K426/08 | GI-19 | KIIc | 2008 | HM486955 |  |
| K33/09 | GI-19 | KIIc | 2009 | HM486956 |  |
| K40/09 | GI-19 | KIIc | 2009 | HM486957 |  |
| K74/09 | GI-19 | KIIc | 2009 | HM486958 |  |
| K344/09 | GI-19 | KIIc | 2009 | HM486959 |  |
| K88/10 | GI-19 | KIIc | 2010 | HM486960 |  |
| K147/10 | GI-19 | KIIc | 2010 | HM486961 |  |
| K716/05 | GI-19 | KIIc | 2005 | HM486962 |  |
| K23/10 | GVI-1 | - | 2010 | JF804677 |  |
| K26/10 | GVI-1 | - | 2010 | JF804678 |  |
| K46/10 | GVI-1 | - | 2010 | JF804679 |  |
| K119/09 | GVI-1 | - | 2009 | JF804680 |  |
| K194/10 | GI-19 | KIIc | 2010 | JF804681 |  |
| K201/10 | GI-19 | KIIc | 2010 | JF804682 |  |
| K226/10 | GI-19 | KIIc | 2010 | JF804683 |  |
| K235/10 | GI-19 | KIIc | 2010 | JF804684 |  |
| K236/10 | GI-19 | KIIc | 2010 | JF804685 |  |
| K245/10 | GI-19 | KIIc | 2010 | JF804686 |  |
| K273/09 | GVI-1 | - | 2009 | JF804687 |  |
| K307/10 | GI-19 | KIIc | 2010 | JF804688 |  |
| K308/09 | GVI-1 | - | 2009 | JF804689 |  |
| K026-10 | GVI-1 | - | 2010 | JQ693048 | (106) |
| K2 | GI-19 | KIIa | 2011 | JQ920378 | (105) |
| 1038 | GI-19 | KIIc | 2010 | JQ920379 |  |
| 1043 | GI-19 | KIIc | 2010 | JQ920380 |  |
| 1107 | GI-19 | KIId | 2010 | JQ920381 |  |
| 1110 | GI-19 | KIIc | 2010 | JQ920382 |  |
| 1114 | GI-19 | KIId | 2010 | JQ920383 |  |
| 1115 | GI-19 | KIIc | 2010 | JQ920384 |  |
| 1116 | GI-19 | KIIc | 2010 | JQ920385 |  |
| 1123 | GI-19 | KIIa | 2010 | JQ920386 |  |
| 8065 | GI-19 | KIId | 2008 | JQ920387 |  |
| 9011 | GI-15 | - | 2009 | JQ920389 |  |
| 9106 | GI-19 | KIIc | 2009 | JQ920390 |  |
| 9137-5 | GI-19 | KIIc | 2009 | JQ920391 |  |
| 9137-6 | GI-19 | KIIc | 2009 | JQ920392 |  |
| 9137-7 | GI-19 | KIIc | 2009 | JQ920393 |  |
| 9137-8 | GI-19 | KIIc | 2009 | JQ920394 |  |
| 9138 | GI-19 | KIIc | 2009 | JQ920395 |  |
| 11026 | GI-19 | KIId | 2011 | JQ920396 |  |
| 11031 | GI-1 | - | 2011 | JQ920397 |  |
| 11035 | GI-19 | KIId | 2011 | JQ920398 |  |
| 11036 | GI-19 | KIId | 2011 | JQ920399 |  |
| 11038 | GI-19 | KIId | 2011 | JQ920400 |  |
| 11039 | GI-19 | KIId | 2011 | JQ920401 |  |
| 11044 | GI-19 | KIIa | 2011 | JQ920402 |  |
| 11045 | GI-19 | KIId | 2011 | JQ920403 |  |
| 11051 | GI-19 | KIIa | 2011 | JQ920404 |  |
| 11052 | GI-1 | - | 2011 | JQ920405 |  |
| QIA-03342 | GI-19 | KIIb | 2003 | KU900739 | (42) |
| 10043 | GI-19 | KIIc | 2010 | KU900743 |  |
| QIA-q43 | GI-19 | KIIb | 2006 | KU900744 |  |
| KrD1515 | GVI-1 | GVI-1 | 2015 | MF176212 | (107) |
| K046-12 | GI-1 | - | 2012 | MK618758 | (108) |
| K047-12 | GI-19 | KIId | 2012 | MK618759 |  |
| IBV/Korea/151/2020 | GI-15 | - | 2020 | MW877606 | (43) |
| IBV/Korea/49/2020 | GI-15 | - | 2020 | MW877607 |  |
| IBV/Korea/189/2017 | GI-15 | - | 2017 | MW877608 |  |
| IBV/Korea/48/2020 | GI-15 | - | 2020 | MW877609 |  |
| IBV/Korea/40/2020 | GI-19 | KIId | 2020 | MW877610 |  |
| IBV/Korea/68/2020 | GI-19 | KIId | 2020 | MW877611 |  |
| IBV/Korea/224/019 | GI-19 | KIId | 2019 | MW877612 |  |
| IBV/Korea/63/2020 | GI-19 | KIId | 2020 | MW877613 |  |
| IBV/Korea/77/2020 | GI-19 | KIId | 2020 | MW877614 |  |
| IBV/Korea/95/2020 | GI-19 | KIId | 2020 | MW877615 |  |
| IBV/Korea/111/2020 | GI-19 | KIId | 2020 | MW877616 |  |
| IBV/Korea/115/2020 | GI-19 | KIId | 2020 | MW877617 |  |
| IBV/Korea/96/2020 | GI-19 | KIId | 2020 | MW877618 |  |
| IBV/Korea/135/2019 | GI-19 | KIId | 2019 | MW877619 |  |
| IBV/Korea/84/2020 | GI-19 | KIId | 2020 | MW877620 |  |
| IBV/Korea/25/2020 | GI-19 | KIId | 2020 | MW877621 |  |
| IBV/Korea/46/2020 | GI-19 | KIId | 2020 | MW877622 |  |
| IBV/Korea/73/2020 | GI-19 | KIId | 2020 | MW877623 |  |
| IBV/Korea/108/2019 | GI-19 | KIId | 2019 | MW877624 |  |
| IBV/Korea/40/2019 | GI-19 | KIId | 2019 | MW877625 |  |
| IBV/Korea/183/2018 | GI-19 | KIId | 2018 | MW877626 |  |
| IBV/Korea/29/2018 | GI-19 | KIId | 2018 | MW877627 |  |
| IBV/Korea/18/2018 | GI-19 | KIId | 2018 | MW877628 |  |
| IBV/Korea/22/2018 | GI-19 | KIId | 2018 | MW877629 |  |
| IBV/Korea/25/2018 | GI-19 | KIId | 2018 | MW877630 |  |
| IBV/Korea/61/2018 | GI-19 | KIId | 2018 | MW877631 |  |
| IBV/Korea/76/2019 | GI-19 | KIId | 2019 | MW877632 |  |
| IBV/Korea/150/2019 | GI-19 | KIId | 2019 | MW877633 |  |
| IBV/Korea/144/2018 | GI-19 | KIId | 2018 | MW877634 |  |
| IBV/Korea/151/2018 | GI-19 | KIId | 2018 | MW877635 |  |
| IBV/Korea/151/2019 | GI-19 | KIId | 2019 | MW877636 |  |
| IBV/Korea/193/2018 | GI-19 | KIId | 2018 | MW877637 |  |
| IBV/Korea/60/2018 | GI-19 | KIId | 2018 | MW877638 |  |
| IBV/Korea/23/2018 | GI-19 | KIId | 2018 | MW877639 |  |
| IBV/Korea/24/2018 | GI-19 | KIId | 2018 | MW877640 |  |
| IBV/Korea/28/2018 | GI-19 | KIId | 2018 | MW877641 |  |
| IBV/Korea/59/2016 | GI-19 | KIId | 2016 | MW877642 |  |
| IBV/Korea/064/2018 | GI-19 | KIId | 2018 | MW877643 |  |
| IBV/Korea/17/2018 | GI-19 | KIId | 2018 | MW877644 |  |
| IBV/Korea/63/2016 | GI-19 | KIId | 2016 | MW877645 |  |
| IBV/Korea/37/2017 | GI-19 | KIId | 2017 | MW877646 |  |
| IBV/Korea/76/2017 | GI-19 | KIId | 2017 | MW877647 |  |
| IBV/Korea/113/2017 | GI-19 | KIId | 2017 | MW877648 |  |
| IBV/Korea/33/2017 | GI-19 | KIId | 2017 | MW877649 |  |
| IBV/Korea/85/2016 | GI-19 | KIId | 2016 | MW877650 |  |
| IBV/Korea/87/2016 | GI-19 | KIId | 2016 | MW877651 |  |
| IBV/Korea/4/2016 | GI-19 | KIId | 2016 | MW877652 |  |
| IBV/Korea/73/2016 | GI-19 | KIId | 2016 | MW877653 |  |
| IBV/Korea/166/2016 | GI-19 | KIId | 2016 | MW877654 |  |
| IBV/Korea/98/2016 | GI-19 | KIId | 2016 | MW877655 |  |
| IBV/Korea/80/2016 | GI-19 | KIIa | 2016 | MW877656 |  |
| IBV/Korea/148/2019 | GI-19 | KIIa | 2019 | MW877657 |  |
| IBV/Korea/5/2020 | GI-19 | KIIa | 2020 | MW877658 |  |
| IBV/Korea/97/2016 | GI-19 | KIIa | 2016 | MW877659 |  |
| IBV/Korea/55/2017 | GI-19 | KIIa | 2017 | MW877660 |  |
| IBV/Korea/173/2017 | GI-19 | KIIa | 2017 | MW877661 |  |
| IBV/Korea/177/2018 | GI-19 | KIIa | 2018 | MW877662 |  |
| IBV/Korea/51/2018 | GI-19 | KIIa | 2018 | MW877663 |  |
| IBV/Korea/62/2018 | GI-19 | KIIa | 2018 | MW877664 |  |
| IBV/Korea/181/2020 | GI-19 | KIIc | 2020 | MW877665 |  |
| IBV/Korea/264/2019 | GI-19 | KIIc | 2019 | MW877666 |  |
| IBV/Korea/269/2019 | GI-19 | KIIc | 2019 | MW877667 |  |
| IBV/Korea/163/2021 | GI-15 and GI-19 recombinant | - | 2021 | OP886679 | (110) |
| IBV/Korea/289/2019 | GI-15 and GI-19 recombinant |  | 2019 | OP886678 |  |
| IBV/Korea/17/2018 | GI-19 | KIId | 2018 | OR050543 | (104) |
| IBV/Korea/25/2020 | GI-19 | KIId | 2020 | OR050544 |  |
| IBV/Korea/37/2017 | GI-19 | KIId | 2017 | OR050545 |  |
| IBV/Korea/55/2011 | GI-19 | KIId | 2011 | OR050546 |  |
| IBV/Korea/61/2018 | GI-19 | KIId | 2018 | OR050547 |  |
| IBV/Korea/63/2020 | GI-19 | KIId | 2020 | OR050548 |  |
| IBV/Korea/68/2020 | GI-19 | KIId | 2020 | OR050549 |  |
| IBV/Korea/76/2017 | GI-19 | KIId | 2017 | OR050550 |  |
| IBV/Korea/77/2020 | GI-19 | KIId | 2020 | OR050551 |  |
| IBV/Korea/87/2016 | GI-19 | KIId | 2016 | OR050552 |  |
| IBV/Korea/99/2016 | GI-19 | KIId | 2016 | OR050553 |  |
| IBV/Korea/150/2019 | GI-19 | KIIc | 2019 | OR050554 |  |
| IBV/Korea/193/2018 | GI-19 | KIId | 2018 | OR050555 |  |
| IBV/Korea/307/2010 | GI-19 | KIIc | 2010 | OR050556 |  |
| IBV/Korea/415/2010 | GI-19 | KIId | 2010 | OR050557 |  |
| IBV/Korea/443/2010 | GI-19 | KIIa | 2010 | OR050558 |  |
| IBV/Korea/40/2019 | GI-19 | KIId | 2019 | OR050559 |  |
| IBV/Korea/85/2016 | GI-19 | KIId | 2016 | OR050560 |  |
| IBV/Korea/98/2016 | GI-19 | KIIa | 2016 | OR050561 |  |
| IBV/Korea/80/2016 | GI-19 | KIIa | 2016 | OR050562 |  |
| IBV/Korea/111/2020 | GI-19 | KIId | 2020 | OR050563 |  |
| IBV/Korea/40/2020 | GI-19 | KIId | 2020 | OR050564 |  |
| IBV/Korea/109/2011 | GI-11 | - | 2011 | PQ178996 | (44) |
| Kr/D342/03 | GI-19 | K-IIb | 2003 | PQ431471 | (34) |
| Kr/D343/03 | GI-19 | K-IIb | 2003 | PQ431472 |  |
| Kr/D354/03 | GI-19 | K-IIb | 2003 | PQ431473 |  |
| Kr/D379/03 | GI-19 | K-IIb | 2003 | PQ431474 |  |
| Kr/D23/13 | GI-19 | K-IIa | 2013 | PQ431475 |  |
| Kr/Q22/13 | GI-19 | K-IId | 2013 | PQ431476 |  |
| Kr/D89/15 | GI-19 | K-IIa | 2015 | PQ431477 |  |
| Kr/D41/16 | GI-19 | K-IIa | 2016 | PQ431478 |  |
| Kr/Q23/17 | GI-19 | K-IIb | 2017 | PQ431479 |  |
| Kr/D41/18 | GI-19 | K-IId | 2018 | PQ431480 |  |
| Kr/D42/18 | GI-19 | K-IId | 2018 | PQ431481 |  |
| Kr/N101/18 | GI-19 | K-IId | 2018 | PQ431482 |  |
| Kr/N143/18 | GI-19 | K-IId | 2018 | PQ431483 |  |
| Kr/N288/18 | GI-19 | K-IId | 2018 | PQ431484 |  |
| Kr/N49/18 | GI-19 | K-IId | 2018 | PQ431485 |  |
| Kr/D30/19 | GI-19 | K-IIa | 2019 | PQ431486 |  |
| Kr/D60/19 | GI-19 | K-IId | 2019 | PQ431487 |  |
| Kr/D68/19 | GI-19 | K-IId | 2019 | PQ431488 |  |
| Kr/Q10/19 | GI-19 | K-IId | 2019 | PQ431489 |  |
| Kr/D42/20 | GI-19 | K-IIc | 2020 | PQ431490 |  |
| Kr/D39/21 | GI-19 | K-IId | 2021 | PQ431491 |  |
| Kr/D60/21 | GI-19 | K-IId | 2021 | PQ431492 |  |
| Kr/N28/21 | GI-19 | K-IIa | 2021 | PQ431493 |  |
| Kr/Q6/21 | GI-19 | K-IIc | 2021 | PQ431494 |  |
| Kr/N34/21 | GI-19 | K-IId | 2021 | PQ431495 |  |
| Kr/N48/21 | GI-19 | K-IId | 2021 | PQ431496 |  |
| Kr/D72/22 | GI-19 | K-IId | 2022 | PQ431497 |  |
| Kr/D29/22 | GI-19 | K-IId | 2022 | PQ431498 |  |
| Kr/N102/22 | GI-19 | K-IId | 2022 | PQ431499 |  |
| Kr/N120/22 | GI-19 | K-IId | 2022 | PQ431500 |  |
| Kr/N238/22 | GI-19 | K-IIc | 2022 | PQ431501 |  |
| Kr/N259/22 | GI-19 | K-IId | 2022 | PQ431502 |  |
| Kr/N44/22 | GI-19 | K-IId | 2022 | PQ431503 |  |
| Kr/N99/22 | GI-19 | K-IId | 2022 | PQ431504 |  |
| Kr/D39/23 | GI-19 | K-IId | 2023 | PQ431505 |  |
| Kr/D61/23 | GI-19 | K-IId | 2023 | PQ431506 |  |
| Kr/D86/23 | GI-19 | K-IId | 2023 | PQ431507 |  |
| Kr/D90/23 | GI-19 | K-IIa | 2023 | PQ431508 |  |
| Kr/D90/22 | GI-19 | K-IId | 2022 | PQ431509 |  |
| Kr/D35/23 | GI-19 | K-IIe | 2023 | PQ431510 |  |
| Kr/N435/23 | GI-19 | K-IIe | 2023 | PQ431511 |  |
| Kr/N447/23 | GI-19 | K-IIe | 2023 | PQ431512 |  |
| Kr/D73/23 | GI-19 | K-IIe | 2023 | PQ431513 |  |
| Kr/D89/23 | GI-19 | K-IIe | 2023 | PQ431514 |  |
| Kr/N6/24 | GI-19 | K-IIe | 2024 | PQ431515 |  |
| Kr/D2/24 | GI-19 | K-IIe | 2024 | PQ431516 |  |
| Korea/AD21/13 | GI-19 | KIId | 2013 | OP765916 | (33) |
| Korea/AD22/13 | GI-19 | KIId | 2013 | OP765917 |  |
| Korea/AD24/13 | GI-19 | KIId | 2013 | OP765918 |  |
| Korea/AD25/13 | GI-19 | KIId | 2013 | OP765919 |  |
| Korea/AD62/16 | GI-19 | KIId | 2016 | OP765920 |  |
| Korea/AD68/14 | GI-19 | KIId | 2014 | OP765921 |  |
| Korea/AD69/14 | GI-19 | KIId | 2014 | OP765922 |  |
| Korea/AQ22/13 | GI-19 | KIId | 2013 | OP765923 |  |
| Korea/AQ28/13 | GI-19 | KIId | 2013 | OP765924 |  |
| Korea/AQ30/13 | GI-19 | KIId | 2013 | OP765925 |  |
| Korea/D07/17 | GI-19 | KIId | 2017 | OP765926 |  |
| Korea/QIA01507/05 | GI-19 | KIIa | 2005 | OP765928 |  |
| Korea/QIA02044/03 | GI-19 | KIIa | 2003 | OP765929 |  |
| Korea/QIA02058/03 | GI-19 | KIIa | 2003 | OP765930 |  |
| Korea/QIA02117/05 | GI-19 | KIIa | 2005 | OP765931 |  |
| Korea/QIA02142/03 | GI-19 | KIIa | 2003 | OP765932 |  |
| Korea/QIA02157/03 | GI-19 | KIIa | 2003 | OP765933 |  |
| Korea/QIA02210/05 | GI-19 | KIIa | 2005 | OP765934 |  |
| Korea/QIA02292/03 | GI-19 | KIIa | 2003 | OP765935 |  |
| Korea/QIA03031/03 | GI-19 | KIIa | 2003 | OP765936 |  |
| Korea/QIA03343/03 | GI-19 | KIIb | 2003 | OP765937 |  |
| Korea/QIA03354/03 | GI-19 | KIIb | 2003 | OP765938 |  |
| Korea/QIAkr10/04 | GI-19 | KIIa | 2004 | OP765939 |  |
| Korea/QIAkr17/04 | GI-19 | KIIa | 2003 | OP765940 |  |
| Korea/QIAkr162/04 | GI-19 | KIIa | 2004 | OP765941 |  |
| Korea/R29-1/16 | GI-19 | KIId | 2016 | OP765942 |  |
| Korea/SNU11072/11 | GI-19 | KIId | 2011 | OP765943 |  |
| Korea/SNU12083/12 | GI-19 | KIId | 2012 | OP765944 |  |
| Korea/SNU12096/12 | GI-19 | KIId | 2012 | OP765945 |  |
| Korea/SNU12099/12 | GI-19 | KIId | 2012 | OP765946 |  |
| Korea/SNU13033/13 | GI-19 | KIId | 2013 | OP765947 |  |
| Korea/SNU16005/16 | GI-19 | KIId | 2016 | OP765948 |  |
| Korea/SNU16007/16 | GI-19 | KIId | 2016 | OP765949 |  |
| Korea/SNU16015/16 | GI-15 | - | 2016 | OP765950 |  |
| Korea/SNU16043/16 | GI-15 | - | 2016 | OP765951 |  |
| Korea/SNU18006/18 | GI-19 | KIId | 2018 | OP765952 |  |
| Korea/SNU19012/19 | GI-19 | KIId | 2019 | OP765955 |  |
| Korea/SNU19018/19 | GI-19 | KIId | 2019 | OP765956 |  |
| Korea/SNU19020/19 | GI-19 | KIId | 2019 | OP765957 |  |
| Korea/SNU19022/19 | GI-19 | KIId | 2019 | OP765958 |  |
| Korea/SNU19029/19 | GI-19 | KIId | 2019 | OP765959 |  |
| Korea/SNU19032/19 | GI-19 | KIIa | 2019 | OP765960 |  |
| Korea/SNU19048/19 | GI-19 | KIId | 2019 | OP765961 |  |
| Korea/SNU19069/19 | GI-15 | - | 2019 | OP765962 |  |
| Korea/SNU19071/19 | GI-15 | - | 2019 | OP765963 |  |
| Korea/SNU20002/20 | GI-19 | KIIc | 2020 | OP765964 |  |
| Korea/SNU20007/20 | GI-15 | - | 2020 | OP765965 |  |
| Korea/SNU20010/20 | GI-19 | KIIc | 2020 | OP765966 |  |
| Korea/SNU20017/20 | GI-19 | KIId | 2020 | OP765968 |  |
| Korea/SNU20018/20 | GI-19 | KIId | 2020 | OP765969 |  |
| Korea/SNU20020/20 | GI-19 | KIId | 2020 | OP765970 |  |
| Korea/SNU20027/20 | GI-15 | - | 2020 | OP765971 |  |
| Korea/SNU20031-2/20 | GI-15 | - | 2020 | OP765972 |  |
| Korea/SNU20047/20 | GI-19 | KIIc | 2020 | OP765973 |  |
| Korea/SNU20048/20 | GI-19 | KIId | 2020 | OP765974 |  |
| Korea/SNU20049/20 | GI-19 | KIId | 2020 | OP765975 |  |
| Korea/SNU20050/20 | GI-19 | KIIc | 2020 | OP765976 |  |
| Korea/SNU21001/21 | GI-19 | KIId | 2021 | OP765977 |  |
| Korea/SNU21003/21 | GI-19 | KIId | 2021 | OP765978 |  |
| Korea/SNU21004/21 | GI-15 | - | 2021 | OP765979 |  |
| Korea/SNU21005/21 | GI-19 | KIId | 2021 | OP765980 |  |
| Korea/SNU21011/21 | GI-19 | KIIc | 2021 | OP765981 |  |
| Korea/SNU21012/21 | GI-19 | KIIc | 2021 | OP765982 |  |
| Korea/SNU21013/21 | GI-19 | KIIc | 2021 | OP765983 |  |
| Korea/SNU11033/11 | GI-19 | KIIa | 2011 | OP765984 |  |
| Korea/SNU11063/11 | GI-19 | KIIa | 2011 | OP765985 |  |
| Korea/SNU11081/11 | GI-19 | KIIa | 2011 | OP765986 |  |
| Korea/SNU12047/12 | GI-19 | KIIa | 2012 | OP765987 |  |
| Korea/AD53/13 | GI-19 | KIIa | 2013 | OP765988 |  |
| Korea/AD23/13 | GI-19 | KIIa | 2013 | OP765989 |  |
| Korea/AD19/13 | GI-19 | KIIa | 2013 | OP765990 |  |
| Korea/14026/14 | GI-19 | KIIa | 2014 | OP765991 |  |
| Korea/AD65/16 | GI-19 | KIIa | 2016 | OP765992 |  |
| Korea/D32/15 | GI-19 | KIIa | 2015 | OP765993 |  |
| Korea/D05/15 | GI-19 | KIIa | 2015 | OP765994 |  |
| Korea/15020/15 | GI-19 | KIIa | 2015 | OP765995 |  |
| Korea/D44/15 | GI-19 | KIIa | 2015 | OP765996 |  |
| Korea/D16/16 | GI-19 | KIIa | 2016 | OP765997 |  |
| Korea/16D62/16 | GI-19 | KIIa | 2016 | OP765998 |  |
| Korea/D147/2/16 | GI-19 | KIIa | 2016 | OP765999 |  |
| Korea/D90/16 | GI-19 | KIIa | 2016 | OP766000 |  |
| Korea/SNU19050/19 | GI-19 | KIIa | 2019 | OP766001 |  |
| Korea/SNU19063/19 | GI-19 | KIIa | 2019 | OP766002 |  |
| BP-CaKII | GI-19 | KIIa | 2011 | MF924724 | (16) |
| IBV24-7 | GX-1 | - | 2024 | PV920038 |  |
| IBV24-12 | GI-19 | KIId | 2024 | PV920039 |  |
| IBV24-13 | GI-19 | KIIc | 2024 | PV920040 |  |
| IBV24-22 | GI-19 | KIIc | 2024 | PV920041 |  |
| IBV24-23 | GI-19 | KIId | 2024 | PV920042 |  |
| IBV24-24 | GI-19 | KIId | 2024 | PV920043 |  |
| IBV24-25 | GI-19 | KIId | 2024 | PV920044 |  |
| IBV24-26 | GI-19 | KIId | 2024 | PV920045 |  |
| IBV24-27 | GX-1 | - | 2024 | PV920046 |  |
| IBV24-28 | GI-19 | KIIa | 2024 | PV920047 |  |
| IBV-BI24-12 | GI-19 | KIIc | 2024 | PV920048 |  |
| IBV-BI24-13 | GI-19 | KIIc | 2024 | PV920049 |  |
| IBV-BI24-14 | GI-19 | KIIc | 2024 | PV920050 |  |
| IBV-BI24-17 | GI-19 | KIIc | 2025 | PV920051 |  |
| IBV-BI25-3 | GI-19 | KIId | 2025 | PV920052 |  |
| IBV-BI25-4 | GI-19 | KIId | 2025 | PV920053 |  |
| IBV-BI25-5 | GX-1 | - | 2025 | PV920054 |  |
| IBV-BI25-6 | GI-19 | KIId | 2025 | PV920055 |  |
| IBV-BI25-7 | GI-19 | KIId | 2025 | PV920056 |  |
| IBV-SL25-2 | GI-19 | KIIe | 2025 | PV920057 |  |
